# Prophylactic Bilateral Salpingectomy as a Prevention Strategy in Women at High-Risk of Ovarian Cancer: A Mini-Review

**DOI:** 10.3389/fonc.2014.00021

**Published:** 2014-02-10

**Authors:** Tess Schenberg, Gillian Mitchell

**Affiliations:** ^1^The Western Health Public Hospital, Melbourne, VIC, Australia; ^2^Peter MacCallum Cancer Centre, Melbourne, VIC, Australia; ^3^Sir Peter MacCallum Cancer Centre Department of Oncology, University of Melbourne, Melbourne, VIC, Australia

**Keywords:** BRCA, bilateral salpingectomy, ovarian cancer, tubal cancer, bilateral salpingo-oophorectomy, cancer prevention

## Abstract

Risk-reducing bilateral salpingo-oophorectomy is a proven strategy to reduce the risk of serous ovarian cancer associated with germline *BRCA* mutations. It is most effective when performed before natural menopause, but it will render a woman prematurely menopausal. The tubal hypothesis of serous ovarian cancer brings with it the possibility of the alternative surgical approach in younger women comprising of risk-reducing bilateral salpingectomy while conserving their ovaries until nearer the age of natural menopause, when a delayed bilateral oophorectomy can be performed. This article will review the evidence behind the tubal hypothesis of serous ovarian cancer and explore the opportunities for translating this into clinical cancer prevention practice.

Women at a high-risk of developing serous ovarian cancer due to their inheritance of a germline mutation in a cancer predisposition gene, such as *BRCA1, BRCA2* (1), are strongly advised to have prophylactic surgery to remove their ovaries and fallopian tubes (risk-reducing bilateral salpingo-oophorectomy RRBS0) once childbearing is complete (2, 3). Screening for ovarian cancer in high-risk populations is not recommended although a recent report suggests a degree of tumor down-staging with a strict adherence to an intensive screening protocol (4). No mortality benefit has been shown for ovarian cancer screening, even with strict adherence to screening protocols, in contrast to a clear mortality reduction with RRBSO in this population (5). Premenopausal BSO also brings a 50% reduction in breast cancer incidence in this high-risk group (5) reinforcing the recommendation for early RRBSO.

The timing of an RRBSO is crucial as the stakes are high. On one hand there is the risk of death from cancer, but this needs to be balanced by the potential for significant morbidity and occasional mortality as a consequence of the procedure itself. These are often young patients without cancer and if guided to the wrong prophylactic strategy, they could develop invasive and potentially incurable cancer. However, the risks of the procedure itself also need to be considered, including the immediate surgical and anesthetic risks but also the medical and psychological complications of plunging a woman into a premature menopause.

RRBSO can be a morbid procedure, particularly for younger premenopausal women, although the majority report a positive outcome overall (6–8). In the non-high-risk population, a bilateral oophorectomy at a younger age is associated with increased all-cause mortality (9, 10), predominantly related to the increased risk of cardiovascular disease (11). Reportedly, there is also an increased risk of Parkinsonism, cognitive impairment or dementia (12–14), and osteoporosis (15). While there are good prospective data to support a short-term improvement in mortality for RRBSO in high-risk women (5), the very long-term effects on morbidity and mortality in this group are unknown (6). Obviously, any option to prevent women experiencing an early menopause is going to be attractive to both clinicians and patients. Since the tubal hypothesis of ovarian cancer was first published in 2007 (16), there has been increasing discussion about a staged approach of initial bilateral salpingectomy (RRBS) once childbearing is complete, followed by a delayed oophorectomy (RRBO) closer to natural menopause (17–19).

Prior to 2001, the hypothesis underlying the pathogenesis of ovarian cancer implicated the ovarian surface epithelium or cortical epithelial inclusions that occur during ovulation, with the different ovarian cancer subtypes due to cellular metaplasia. Once initiated the ovarian cancer would then spread to the fallopian tube and other gynecological organs and the wider pelvic and abdominal cavities. In 2001, reports of a high rate of tubal neoplastic lesions in the RRBSO specimens from high-risk women were published (20, 21). In these reports, fallopian tubes of high-risk women were carefully examined and preinvasive cancerous lesions were found leading to other reports with similar findings (22, 23) and the unifying hypothesis by Crum et al. suggesting that the fallopian tubes were the site of origin of many serous ovarian cancers (16). These precursor lesions – tubal intraepithelial carcinomas (TICs) – had no correlating precursor lesions within the ovary. When specimens from women with serous ovarian cancers, untested for *BRCA* mutations, were examined these lesions were also found in at least 40–60% of cases and the fimbrial end of the fallopian tube obliterated in another 20% (24, 25). Further support of the tubal origin hypothesis came from the highly similar cytological features and striking molecular similarities between TICs and invasive high grade ovarian cancers (25). These include identical *TP53* mutations, a high proliferation rate, chromosomal instability, and gene expression profiles, which all support a clonal origin (26–28).

A refinement to the tubal hypothesis is that the fimbrial ends of the tubes appear to be most vulnerable to malignant transformation, which may explain why tubal ligation provides some ovarian cancer protection in *BRCA* mutation carriers as well as women in the general population (29). In 2006, researchers from Boston described a protocol for sectioning and extensively examining the fimbrial end of the fallopian tubes (SEE-FIM). The fimbria were an area of interest as they are exposed to the peritoneal cavity, are in close proximity to the ovarian surface, merge with the serosal mesothelium, and often contain transitional metaplasia (26). It was found, and subsequently confirmed by others using the same sectioning technique, that the fimbria were the most common place for precancerous and non-invasive malignant precursor lesions within the fallopian tubes (26, 30, 31). Molecular analyses confirm these observations; within the non-neoplastic mucosa of the distal tubes was a benign precursor entity consisting of foci of strong p53 immunostaining (indicative of a *TP53* mutation), subsequently termed the “p53 signature.” The p53 signature was equally common in non-neoplastic tubes from *BRCA* mutation carriers and controls, but was observed more frequently and was multifocal in fallopian tubes that also contained TIC. Like the prior studies of TIC, p53 signatures predominated in the fimbriae (23, 30). However, despite the predilection for the fimbriae, approximately one-third of TIC lesions have been observed elsewhere in the tube reinforcing the need for total removal of the tube for risk-reducing purposes (32).

From these data a plausible biological model for the pathogenesis of what might be a large proportion of high grade serous ovarian carcinoma has emerged. The hypothesized pathway begins with areas of non-neoplastic distal fallopian tubes developing *TP53* mutations. The hypothesis then suggests that this leads to a non-invasive malignancy that eventually dedifferentiates into invasive malignancy that subsequently implants into the ovary. A prospective review of RRBSO specimens from women at high-risk of ovarian cancer due to their family history or known *BRCA* mutations is supportive of this hypothesis. Of 360 RRBSO specimens reviewed, four invasive malignancies and four TICs were identified – all of which were associated with the tubal epithelium (33).

Clearly this is a compelling theory with a persuasive, although still incomplete, body of evidence behind it and could provide a rationale for risk-reducing bilateral salpingectomy (RRBS). However, it may not be the only route for the pathogenesis of ovarian cancer because the timeframe of the pathogenic process and the point of transfer of malignant or potentially malignant tubal cells to the ovary are not known. It is clear that even when utilizing the FEE-SIM protocol to examine RRBSO specimens there are still ovarian cancers identified that are not associated with any obvious fallopian tube malignancy/pre-malignant lesion. It may be that the tubal primary is too small to be found and/or that another, intra-ovarian, pathway also leads to ovarian cancer and/or that the tubal cells can be transferred to the ovary at a much earlier time point. For example, it may be that during ovulation cortical inclusion cysts are formed incorporating normal tubal epithelial cells (endosalpingiosis), which can then cause carcinoma with an underlying molecular signature consistent with the fallopian tubes (25). If any of these additional theories are correct then high-risk women may be done a serious disservice by neglecting to perform an oophorectomy with salpingectomy.

The evidence supporting the tubal hypothesis of ovarian cancer has already led to calls for bilateral salpingectomy to be added to hysterectomies performed for benign reasons in women at average population risk of ovarian cancer. This was first proposed in 2009 by Salvador et al (34) and has led to a 20× increase in salpingectomy with hysterectomy in Canada (25) although there are still barriers to its routine implementation (35–37). Adding salpingectomy to hysterectomy does not appear to have any immediate increase in complications (38). Additional proposals to perform salpingectomy rather than tubal ligation for women seeking permanent contraception have also been proposed (25).

While the tubal hypothesis is an intriguing one and can be easily integrated into routine care of women at population risk of ovarian cancer requiring hysterectomy or contraception, is the risk:benefit balance tipped in favor of a staged RRBS followed by risk-reducing bilateral oophorectomy (RRBO) at a later date in younger women at high-risk of ovarian cancer? A Canadian group has developed a Markov Monte Carlo simulation model to compare three strategies for risk reduction in women with *BRCA* mutations: (1) RRBSO; (2) RRBS; and (3) RRBS with delayed RRBO (18). The model estimated the number of future breast and ovarian cancers and cardiovascular deaths attributed to premature menopause with each strategy. RRBSO was the most effective risk-reducing strategy but RRBS with delayed RRBO was still cost effective for those women unwilling to have a RRBSO.

Despite the evidence presented above, unfortunately the point has not yet been reached where the tubal hypothesis of ovarian cancer can be reliably used to guide decision-making around prophylactic surgery in high-risk women (39). To safely change current recommendations, we need prospective evidence that the strategy of a staged approach is not inferior to upfront RRBSO. A randomized controlled trial comparing these strategies is unfortunately not feasible. The difficulties inherent in this approach are obvious, recruiting from a highly selected group of patients will take an international effort over many years in order to give sufficient statistical power to detect a state of non-inferiority, but there is also the ethical dilemma for clinicians offering randomization to an untested procedure against one, which has proven mortality benefits in a young population – would enough clinicians be in clinical equipoise in order to recruit sufficient numbers of participants? A prospective cohort study following high-risk women selecting RRBS over RRBSO (risk-reducing bilateral salpingo-oophorectomy) is a more practicable approach to answer the question but would still require a large population to give a statistically significant result. It is unlikely that a single international cohort study will be proposed and funded to answer this question but there are a number of prospective cohort studies in *BRCA* mutation carriers across the world that could provide the necessary outcome data in the future provided the required data can be collected systematically. Furthermore, many familial cancer clinics follow up mutation carriers and would also be in a position to contribute prospective outcome data in the future. Provided that all of these groups can be brought together to pool data, an answer may be forthcoming.

So, what to advise a young *BRCA* mutation carrier who has completed her family while still in her 30s, or is in her 40s and declines RRBSO? Careful counseling is necessary to ensure that she is fully informed about the range of surgical prevention options, explaining the risks, and benefits, of all surgical approaches. It is necessary to emphasize the known mortality and breast cancer risk reduction benefits of RRBSO, and ensure that she is aware of the range of strategies to manage any sequelae arising from a premature surgical menopause. The advantage of the alternative of a staged procedure starting with bilateral salpingectomy then a bilateral oophorectomy at or approaching the age of natural menopause is that it avoids morbidity of premature menopause but this comes at the cost of uncertain impact on overall mortality, ovarian cancer-specific mortality and abrogation, or complete loss of breast cancer risk reduction. The Markov model (18) concluding that RRBS with delayed RRBO salpingectomy followed by delayed oophorectomy yields the highest quality-adjusted life expectancy (18) is intriguing, however, it is essential for a fully informed decision that it is made clear to the high-risk woman that no prospective data yet exists on the efficacy of bilateral salpingectomy in reducing mortality in high-risk women. However, in the end, it is a woman’s decision based on her own preferences and life experiences and it is the role of her medical team to support her in her choices in order to maximize their benefit and minimize their risk. Some prophylactic surgery in the form of bilateral salpingectomy is probably better than no surgery in this high-risk population.

## Author Contributions

Tess Schenberg and Gillian Mitchell, article concept, article drafting, and final approval of manuscript.

## Conflict of Interest Statement

The authors declare that the research was conducted in the absence of any commercial or financial relationships that could be construed as a potential conflict of interest.
